# The Host-Encoded Heme Regulated Inhibitor (HRI) Facilitates Virulence-Associated Activities of Bacterial Pathogens

**DOI:** 10.1371/journal.pone.0068754

**Published:** 2013-07-10

**Authors:** Niraj Shrestha, Justin Boucher, Wael Bahnan, Emily S. Clark, Roland Rosqvist, Kenneth A. Fields, Wasif N. Khan, Kurt Schesser

**Affiliations:** 1 Department of Microbiology and Immunology, University of Miami Miller School of Medicine, Miami, Florida, United States of America; 2 Department of Molecular Biology, Umeå University, Umeå, Sweden; University of Illinois at Chicago College of Medicine, United States of America

## Abstract

Here we show that cells lacking the heme-regulated inhibitor (HRI) are highly resistant to infection by bacterial pathogens. By examining the infection process in wild-type and HRI null cells, we found that HRI is required for pathogens to execute their virulence-associated cellular activities. Specifically, unlike wild-type cells, HRI null cells infected with the gram-negative bacterial pathogen *Yersinia* are essentially impervious to the cytoskeleton-damaging effects of the Yop virulence factors. This effect is due to reduced functioning of the *Yersinia* type 3 secretion (T3S) system which injects virulence factors directly into the host cell cytosol. Reduced T3S activity is also observed in HRI null cells infected with the bacterial pathogen *Chlamydia* which results in a dramatic reduction in its intracellular proliferation. We go on to show that a HRI-mediated process plays a central role in the cellular infection cycle of the Gram-positive pathogen *Listeria* . For this pathogen, HRI is required for the post-invasion trafficking of the bacterium to the infected host cytosol. Thus by depriving *Listeria* of its intracellular niche, there is a highly reduced proliferation of *Listeria* in HRI null cells. We provide evidence that these infection-associated functions of HRI (an eIF2α kinase) are independent of its activity as a regulator of protein synthesis. This is the first report of a host factor whose absence interferes with the function of T3S secretion and cytosolic access by pathogens and makes HRI an excellent target for inhibitors due to its broad virulence-associated activities.

## Introduction

Greater knowledge of the mechanisms employed by microbial pathogens to overcome host defenses has allowed for the development of drug-like molecules that specifically target these pathogen virulence-associated structures. Since at least some of these virulence-associated structures are widely conserved among pathogens of animals and plants, such ‘virulence blocker’ compounds provide an attractive alternative to conventional antibiotics that typically target structures (e.g., ribosomes) or processes (e.g., cell wall synthesis) found in both pathogens as well as members of the microbiota. Several examples of broad-acting small molecule virulence blockers were originally identified as inhibitors of the type 3 secretion (T3S) system of the pathogenic yersiniae which delivers virulence factors directly into the host cell cytosol [1]. Subsequently it was shown that these compounds also inhibit T3SSs of other Gram-negative pathogens such as *Chlamydia* , *Salmonella*, and *Pseudomonas* [2]. Here we broaden this concept by identifying a host-encoded factor that is required by diverse pathogens to execute their respective cellular infection cycles.

In a yeast-based genetic screen using bacterial virulence factors as probes, we found that the stress-induced eIF2 signaling pathway plays a key role in the intracellular activities of both the *Yersinia* protein kinase A (YpkA) and *Yersinia* outer protein J (YopJ) [3]. In eukaryotes eIF2 signaling mediates the cellular responses to a variety of external and internal stress. Mammalian cells possess four different eIF2α kinases (GCN4, PERK, PKR, and HRI) that are activated by distinct stress conditions including nutritional deprivation (GCN4), endoplasmic reticulum stress (PERK), infection by viral-derived RNA (PKR) and heat/oxidative/heme-induced stresses (HRI). Phosphorylation eIF2α inhibits the formation of active ternary complexes thus leading to a reduction in protein synthesis. Our studies indicated that in yeast cells YpkA activated eIF2 signaling whereas YopJ, in contrast, negatively regulated eIF2 signaling [3].

Although the significance of the YpkA-induced eIF2 signaling during infection remains unknown, we subsequently showed that, like in yeast cells, YopJ negatively regulated eIF2 signaling in *Yersinia* -infected mammalian cells [4]. Additionally, we showed that an intact eIF2 signaling pathway was required for the infection-induced activation of NF-κB and pro-inflammatory cytokine expression [4]. In addition to its role in NF-κB activation and cytokine expression, we unexpectedly found that eIF2 signaling counteracts the host cell invasion of *Yersinia* as well as the intracellular pathogens *Chlamydia* and *Listeria* [4]. Cells that lacked a functional eIF2 pathway were highly invaded by these pathogens indicating that eIF2 signaling is important in protective anti-bacterial responses.

The heme-regulated inhibitor (HRI in humans, Hri in mice) was originally identified as the translation-level regulator (through its eIF2α kinase activity) that couples β-globin synthesis with heme levels during erythropoiesis and has more recently been shown to mitigate oxidative stress during erythroid differentiation [5,6]. HRI is also important for various stress responses in yeast and mammalian cells [7,8]. Here we investigated whether HRI plays a role in host cell infection by microbial pathogens. Unexpectedly, we found that HRI positively regulates specific virulence-related activities of diverse bacterial pathogens. Surprisingly, these HRI effects were independent of its canonical function as a translation regulator via eIF2α and thus identify a novel role for HRI in bacterial pathogenesis.

## Materials and Methods

### Host and pathogen strains

*Hri* and *Pkr* MEFs were provided by Randal J. Kaufman and Joan E. Durbin [4,8], respectively, and Hri knockout mice were generously provided by Jane-Jane Chen [9]. Mice were treated humanely in strict accordance with federal and state government guidelines for the Care and Use of Laboratory Animals of the National Institutes of Health and their use was approved by the University of Miami institutional animal care and use committee (protocols 11-186).

The wild-type *Yersinia pseudotuberculosis* strain YPIII/pIB102 [10] was used except in the translocation assay a mutant YPIII strain (YPIII/pIB29MEKA), in which all 5 of the effector Yop-encoding genes were deleted [11], was transformed with a plasmid encoding a hybrid protein consisting of YopE (residues1-130) and a 40-residue Elk tag [12]. The *Chlamydia trachomatis* LGV-434, serovar L2 and the OVA-expressing *Listeria monocytogenes* strains were obtained from the American Type Culture Collection (ATCC) and DMX Corp. (DMX 09-082), respectively. The GFP-expressing *L. monocytogenes* strains were provided by Daniel Portnoy.

### Infections

Transcript analysis was performed as previously described [4]. *Hri* +/+ and -/- MEFs were seeded on cover-slips and infected the next day. Overnight cultures of the *Y. pseudotuberculosis* strains were diluted to O.D. 0.1 in tissue culture media and subsequently propagated at 26 °C for 2 hrs and then 37 °C for 1 hr to induce expression of its type 3 secretion system prior to infection. Following infection, cells were first fixed with 2% paraformaldehyde in phosphate buffer saline (PBS, pH 7.4) for 30 minutes, then treated with permeabilization buffer (1% saponin and 3% bovine serum albumin in PBS) for 20 mins and then blocking buffer (0.3% bovine serum albumin and 0.1% Tween 20 in PBS) for 1 hour. Actin was visualized with Alexa-fluor-conjugated phalloidins (Molecular Probes), nuclei with DAPI (Molecular Probes) and vacuoles with LAMP1 (BD Bioscience). Cell images were captured with a Zeiss LSM 700 confocal microscope and analyzed using MacBiophotonics ImageJ software. The Yop translocation assay was performed as described [4]. To analyze if inhibition of protein synthesis of eukaryotic cells affected the kinetics of the cytotoxic response caused by the *Y* *. pseudotubercolosis* wild-type strain YPIII/pIB102, either 5 or 25 µg/ml of cycloheximide was added to cultured HeLa cells one hour prior and maintained during the infection. The morphology of the infected cells was analyzed by phase contrast microscopy up to 4 hrs post-infection. The *C. trachomatis* infections were performed as described [4] and initiated by adding the bacteria to cultured cells. Following the infection period, cells were either stained for *C. trachomatis* to microscopically determine direct counts of inclusions or alternatively cells were lysed and the resulting supernatants (which contain infectious *C* *. trancomatis* elementary bodies) were used to infect HeLa cell cultures to determine progeny yield. Quiescent (unstimulated) peritoneal macrophages were harvested from *Hri* +/+ and -/- aged/sex matched mice and seeded on coverslips in serum-containing media for a few hours and then unattached cells were removed. Next day cells were infected with *C. trachomatis* and processed as described above for MEFs.

### Flow cytometry and imagining

For the antigen presentation and cytokine expression analyses, single-cell suspensions of splenocytes were prepared from *Hri* +/+ and -/- aged/sex matched mice, passed through a 70 micron filter and resuspended in serum-containing media. Cells were rested for 1 hr and then infected with either the OVA-expressing *L. monocytogenes* strain or the *Y. pseudotuberculosis* strain. For the OVA experiments, splenocytes were infected for 5 h in the presence of brefeldin A and then stained with anti-CD11c (eBioscience), anti-B220 (BD Bioscience), anti-MHC I Ova (Biolegend), anti-CD11b (BD Bioscience), anti-CD4 (eBioscience), anti-CD8 (Biolengend), F4/80 (Caltag Laboratories), and Live/dead near IR (Invitrogen) in fluorescence-activated cell sorting buffer (PBS containing 2% bovine serum albumin and 0.1% sodium azide) for 30 min at 4 °C and then fixed in 2% paraformaldehyde. A Cytofix/Cytoperm kit (BD-Pharmingen) was used to measure intracellular levels of TNFα. Data were acquired using a BD FACS LSRII flow cytometer (Becton Dickinson, San Jose, CA) and analyzed using FlowJo software (Tree Star, Inc.).

The microscopic-based *L. monocytogenes* infection assays were performed using techniques described above with the additional feature that the GFP-expressing bacteria were directly visualized. For the *L. monocytogenes* proliferation assay, MEFs were seeded in a 48-well plate (10^5^ per well) and the next day infected with 4 x 10^6^ cfus that were prepared from a stationary phase culture grown in brain heart infusium at 32 °C without shaking. After a 1.5 hr attachment period excess bacteria were removed and gentamicin was added at 2 µg/ml to kill non-internalized bacteria. After a 1.5 hr extracellular killing period internalized bacteria were enumerated at various time points by lysing the cells with water and determining the colony forming units (cfu) by plating. 

## Results

### The host cellular factor HRI regulates infection-induced TNFα expression

**Figure 1:**
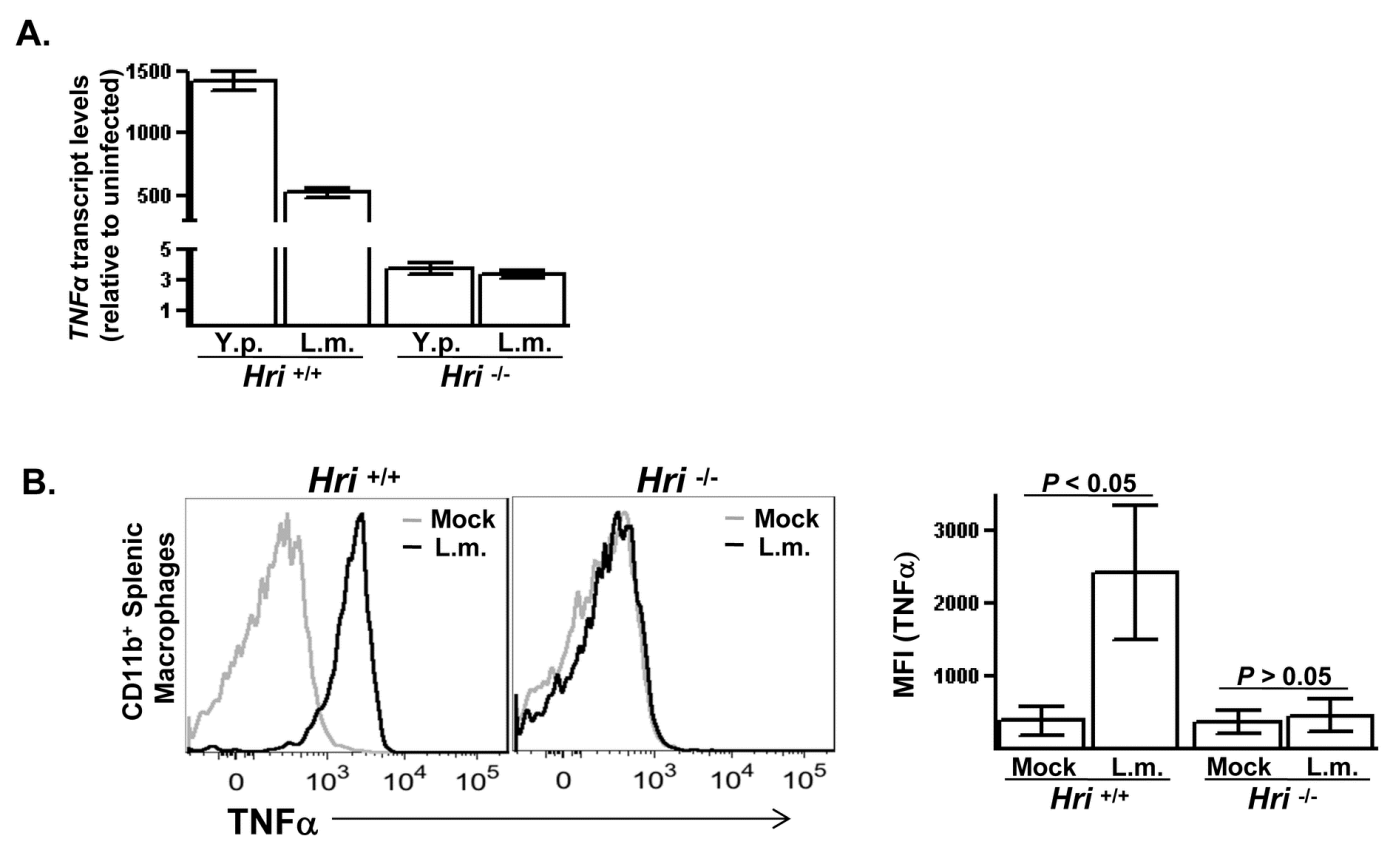
HRI is required for infection-induced cytokine expression. (**A**) Peritoneal macrophages isolated from *Hri* +/+ and -/- mice were infected *in vitro* with either *Yersinia pseudotuberculosis* (Y.p.) or *Listeria monocytogenes* (L.m.) for 3 hr. Plotted are the levels of TNFα-encoding transcripts in infected *Hri* +/+ and -/- cells relative to their respective uninfected control cells. (**B**) Splenocytes isolated from *Hri* +/+ and -/- mice were either left uninfected or infected *in vitro* with L.m. for 5 hrs and then intracellularly stained for TNFα protein. Histrogram displays TNFa levels in macrophages (live, CD11b^+^-gated). The median fluorescence intensities (MFI) of 3 separate experiments is shown (right panel). *P* values calculated using student *t* test.

Prior studies have shown that LPS-induced expression of the proinflammatory cytokine TNFα is reduced in *Hri* -/- compared to *Hri* +/+ macrophages [13]. To determine whether this defective inflammatory response of *Hri* -/- macrophages to LPS reflects a role for HRI in the cellular response to infection with bacterial pathogens, TNFα expression was measured in *Hri* +/+ and *Hri* -/- primary macrophages infected with either *Yersinia pseudotuberculosis* or *Listeria monocytogenes*. In peritoneal macrophages from a *Hri* +/+ mouse infected *in vitro* with these pathogens, TNFα-encoding transcript levels increased >500-fold following a 3-hr infection period (Figure 1A). In contrast, there was only a modest increase (~3-fold) in the levels of TNFα-encoding transcript in infected *Hri* -/- peritoneal macrophages. Consistent with the transcript analysis, there was a several-fold increase in TNFα protein levels in *Hri* +/+ splenic macrophages following 5 hrs of infection with these two pathogens whereas there was essentially no changes in TNFα levels in similarly infected *Hri* -/- macrophages (Figure **1B**; data for *Y. pseudotuberculosis* not shown). Previously we showed that an intact eIF2 signaling pathway was required for infection-induced cytokine expression [4]. The findings presented here suggest that HRI is the primary eIF2α kinase responsible for this effect. However, upon closer examination it became apparent that the role HRI plays in the cellular infection cycle of these pathogens far exceeds its regulation of cytokine expression.

### HRI is required for the function of *Yersinia* T3S system

**Figure 2:**
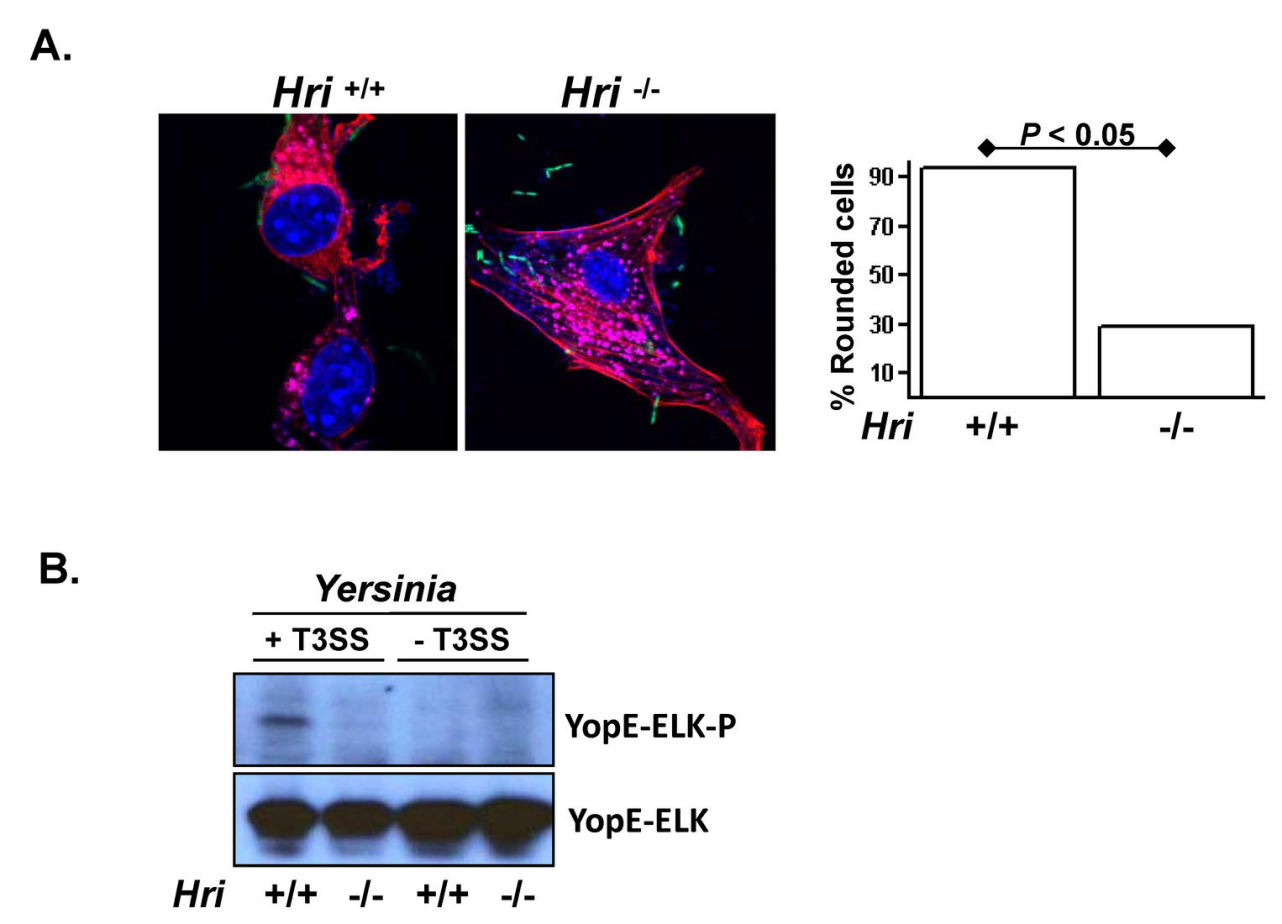
HRI facilitates the translocation of *Yersinia* virulence factors into the cytosol. (**A**) *Hri* +/+ and -/- mouse embryonic fibroblasts (MEFs) were infected with a GFP-expressing strain *Y. pseudotuberculosis* for 2 hrs and then stained for nuclei, vacuoles, and actin (purple, pink, and red, respectively). Plotted is the percentage of rounded cells (resembling the cell shown in the left panel) of at least 5 microscopic fields of approximately 100 total cells. *P* value calculated using student *t* test. (**B**) MEFs were infected with a *Y. pseudotuberculosis* strain deleted for all six Yop effectors and possessing either an intact or a translocation-defective type 3 secretion (T3S) system and harboring a plasmid encoding a YopE-Elk translocation reporter. After 1 hr of infection the resulting whole cell lysates were examined by western analysis using antibodies specific for the Elk epitope tag either in its phosphorylated (YopE-Elk-P) or unphosphorylated (YopE-Elk) forms. YopE-Elk becomes phosphorylated exclusively within the eukaryotic cytosol and therefore the phosphorylation of the Elk epitope is readout for T3S-mediated translocation. For presentation purposes the lanes of a single blot were rearranged.

The pathogenicity of *Yersinia* is inextricably linked to its delivery of virulence factors (or effectors) directly into the host cell cytosol through the T3S system [14]. Upon their delivery into the cytosol, these effectors suppress the induction of host protective cytokine expression via YopJ-dependent mechanisms as well as cause a striking collapse of the cytoskeleton primarily by the actions of YopE [15]. The latter property serves as a readout for T3S activity [16] and can be readily observed in cultured wild-type (*Hri* +/+) mouse embryonic fibroblasts (MEFs) which transition from their normally flatten appearance to a partially detached ‘rounded’ morphology within an hour of infection (Figure 2). Unexpectedly, *Hri* -/- cells displayed almost no overt signs of this cytoskeletal disruption following infection (Figure 2). The resistance of *Hri* -/- cells to T3S-mediated disruption of the cytoskeleton could not be attributed to differential bacterial adhesion as determined both by direct microscopic examination and plating methods (Figure **S1**). Furthermore, there were no discernable generalized defects in the cytoskeletal function in the absence of HRI as both *Hri* +/+ and -/- cells migrated with comparable efficiency in Transwell migration assays (*data not shown*).

To directly test whether the lack of cytoskeletal disruption in *Yersinia* -infected *Hri* -/- cells was due to reduced functioning of the T3S system, we used a YopE-Elk translocation assay as a reporter of the delivery of *Yersinia* virulence factors into the host cell cytosol [12]. Compared to *Hri* +/+ cells, YopE translocated into *Hri* -/- cells was greatly reduected (Figure **2B**; lanes *1* and *2*). The YopE translocation defect in *Hri* -/- MEF cytosol was similar to that observed for a *Yersinia* strain harboring a genetically inactivated T3S system (lanes *3* and *4*; ref. [17]). Additionally, this translocation defect in the *Hri* -/- cells is independent of the Yop virulence factors themselves since this latter assay is performed in a *Yersinia* strain that is genetically deleted for all of the Yop virulence factors. These findings indicate that HRI is required for the T3S-mediated transfer of *Yersinia* -encoded virulence factors into the host cell cytosol. Although host cell processes have been described that promote T3S activity (e.g., the RhoA GTPases [18]), to the best of our knowledge this is the first description of a host factor that, when absent, renders a cell refractory to the translocation of T3S effectors.

To date all of the described activity of HRI is associated with it acting as a regulator of protein synthesis by virtue of its eIF2α kinase activity (see *Introduction*). Previously we reported that protein synthesis rates do not differ between cells infected with wild-type *Y. pseudotuberculosis* versus a T3S mutant derivative [15] and more recently we showed that there were no differences in *Y. pseudotuberculosis* T3S activity between infected wild-type host cells and cells expressing an eIF2α with an S51A replacement (the target residue of the eIF2α kinases) [4]. To further test whether T3S activity and protein synthesis in host cells are functionally linked, we treated cells with cycloheximide prior to (and during) their infection with *Y. pseudotuberculosis*. We observed no differences in either the kinetics or the severity of cytoskeletal disruption between untreated and cycloheximide-treated cells (Figure **S2**). Collectively these findings indicate that function of the *Yersinia* T3S system is not coupled to protein synthesis in host cells (or vice versa). Thus, the mechanism by which HRI positively impacts the T3S system in *Yersinia* infected cells is likely independent of HRI-mediated regulation of protein translation.

### PKR and HRI independently regulate cellular infectivity of *Chlamydia trachomatis*

**Figure 3:**
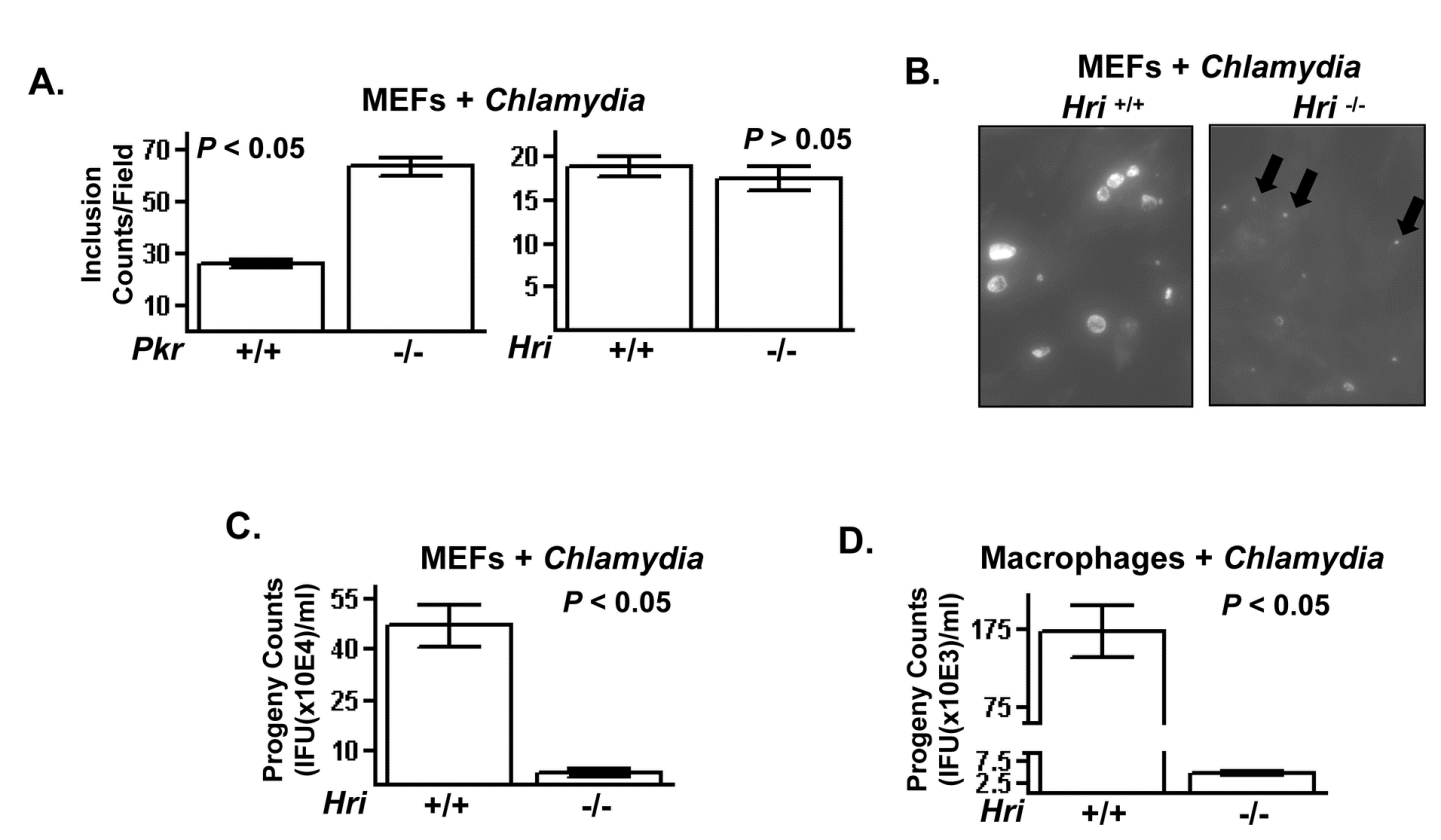
The eIF2α kinase PKR negatively regulates invasion and HRI positively regulates intracellular proliferation of *Chlamydia* . (**A**) *Pkr* +/+ and -/- (left) and *Hri* +/+ and -/- (right) MEFs were infected with *C. trachomatis* L2 for 24 hr and then stained for *C. trachomatis*-containing inclusion bodies. The total number of inclusion forming units is plotted. (**B**) Infected *Hri* +/+ and -/- MEFs are shown with arrows denoting underdeveloped *C* *. tranchomatis* -containing inclusions in the *Hri* -/- cells. (**C**) Cells were infected as described and were then harvested and the resulting lysates used to infect cultured HeLa cells to determine their titer (plotted as ‘progeny counts’). (**D**) Unstimulated peritoneal macrophages were isolated from *Hri* +/+ and -/- mice and infected as described. *P* values calculated using student *t* test.

We recently showed that cells expressing an eIF2α with an S51A replacement (the target residue of the eIF2α kinases) were highly colonized by *C. trachomatis* indicating that this pathway plays a critical role in the complex cellular infection cycle of this bacterial pathogen [4]. Following invasion, *C. trachomatis* forms a membrane-bound inclusion body within which it differentiates from the invasive elementary bodies (EBs) into the proliferating reticulocyte bodies (RBs). To determine whether the eIF2α kinases PKR and/or HRI regulate the infection dynamics of this pathogen, wild-type (+/+) and knockout (-/-) cells were infected with *C. trachomatis* and the number of inclusion bodies (‘direct counts’) were determined. *Pkr* -/- cells were significantly more permissive for invasion compared to *Pkr* +/+ cells (Figure 3A). The higher level of invasion of *Pkr* -/- cells was similar to that we recently reported for cells expressing eIF2α(S51A) [4]. In contrast, there were comparable numbers of inclusion bodies observed in *Hri* +/+ and -/- cells following infection with *C. trachomatis* indicating that HRI does not substantially affect Chlamydial invasion (Figure 3A). The similar invasion phenotypes of the PKR null and eIF2α(S51A) cells suggest that PKR-mediated activation of eIF2 signaling opposes *C. trachomatis* invasion.

Although the number of inclusions that formed in *Hri* +/+ and -/- cells were comparable, there were, however, considerable difference in the sizes of these inclusions. The sizes of the inclusions in the *Hri* -/- cells were much reduced and these inclusions were populated by much fewer *C. trachomatis* compared to the *Hri* +/+ cells (Figure 3B). To quantitatively measure post-invasion proliferation a replating assay is performed in which infected cells are lysed and the yield of infectious units (‘progeny’) is determined. The yield progeny from *Hri* +/+ cells was ~10-fold greater than that recovered from *Hri* -/- cells (Figure 3C). Even greater differences (~50-fold) in progeny yields were observed in *in vitro*-infected peritoneal macrophages isolated from *Hri* +/+ and -/- mice (Figure 3D). These data show that HRI promotes the post-invasion intra-vacuolar proliferation of *C. trachomatis*.

Similar to *Yersinia* , pathogenic *Chlamydia* employs a T3S system to optimally infect eukaryotic cells [19]. The unaffected invasion rate but reduced intra-vacuolar growth phenotype of *C. trachomatis* in *Hri* -/- cells resembles that observed in cells treated with small molecules that disrupt Chlamydial T3SS functioning [20,21]. A high dose infection assay was therefore used to determine whether Chlamydial T3SS activity is reduced in *Hri* -/- cells. A relatively low multiplicity of infection (MOI) was used in the assays shown above such that cells were likely to be infected with a single EB. In cells infected with >1 EBs, each EB initially forms a single inclusion which eventually fuses with other EB-containing inclusions within the same cell. The fusion of multiple inclusions is abrogated by small molecule inhibitors of the T3S system and is dependent on IncA which is an effector of the *C. trachomatis* T3S system [22,23]. Cells were infected with a high MOI of *C. trachomatis* and then examined for the number of inclusions per infected cell. The majority of wild-type infected cells contained a single inclusion whereas *Hri* -/- cells contained small multiple inclusions consistent with a defect in IncA-mediated fusion activity (Figure **S3**). Collectively, these data indicate that HRI plays a positive role in the function of the *C. trachomatis* T3S system.

### HRI is required for efficient trafficking of *Listeria* to the infected cell cytosol

The experiments described above indicate that HRI is required for effectors of the T3S system to gain access to the infected cell cytosol. It was next tested whether HRI regulates the cytosolic access of the Gram-positive pathogen *Listeria monocytogenes*. The intracellular infection cycle of *L. monocytogenes* consist of three distinct phases: (i) host cell invasion; (ii) vacuole escape of the bacterium to the cytosol; and (iii) intracytosolic association with actin and subsequent proliferation; each of these events is mediated by well-characterized virulence factors [24]. Previously we showed that, like *C. trachomatis* noted above, *L. monocytogenes* invaded cells expressing the non-phosphorylatable eIF2α(S51A) at a much higher level compared to wild-type cells [4]. This finding indicated that *L. monocytogenes* functionally interacts with the eIF2 signaling pathway during the cellular invasion phase of its infection cycle.

**Figure 4:**
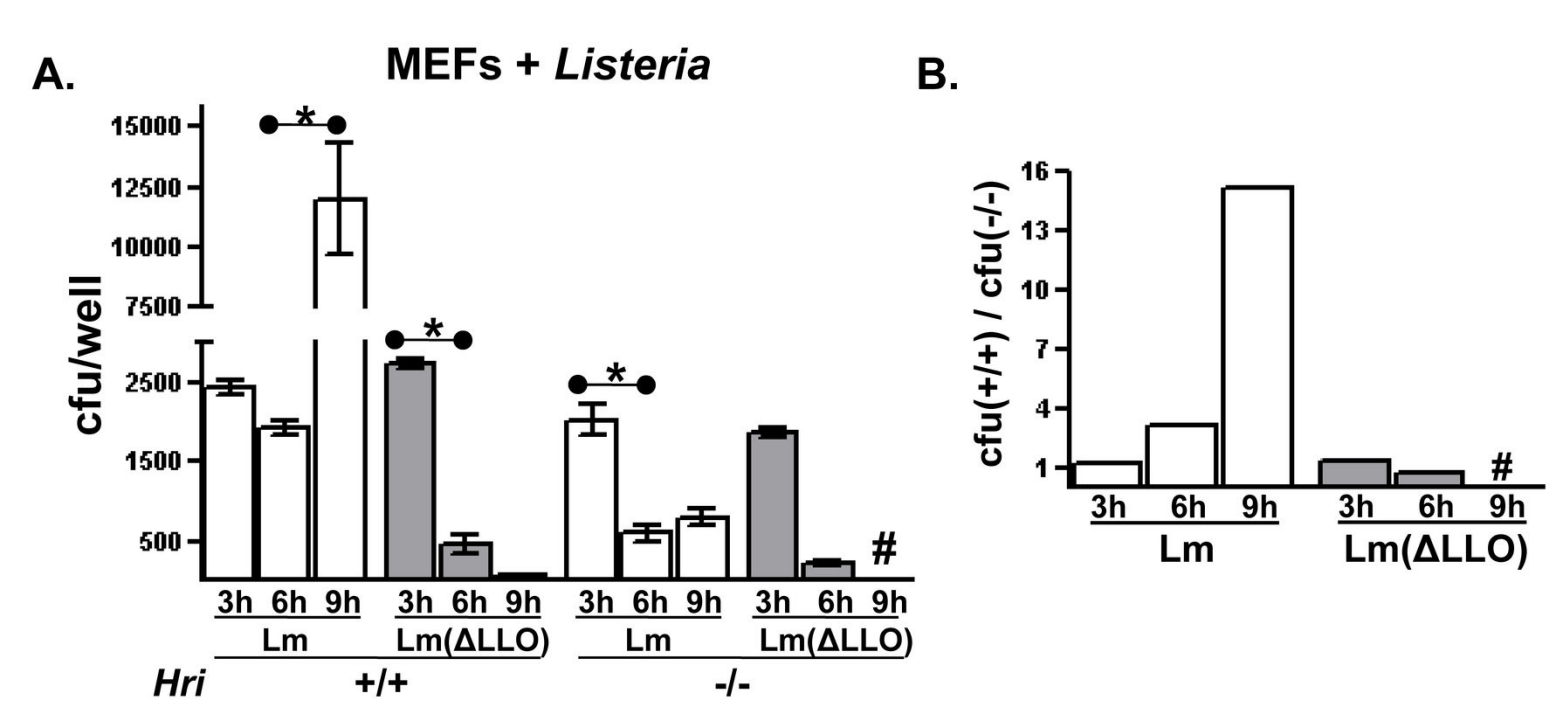
Absence of HRI reduces the intracellular proliferation of *Listeria* . (**A**) Cultured *Hri* +/+ and -/- MEFs were infected with either wild-type *L. monocytogenes* (Lm) or a strain lacking the virulence factor listeriolysin O (ΔLLO). Following a 1.5 hr attachment and invasion period and a 1.5 hr treatment with an antibiotic to kill non-internalized Lm (3 hr total), internalized Lm were enumerated either immediately (3 hr) or at the indicated time points by lysing the cells and determining the colony forming units (cfus) by plating (Using two-tailed student *t* test: *, *P* < 0.05; #, below level of detection). (**B**) Derived from the data shown in (**A**), the ratio between cfus recovered from *Hri* +/+ and -/- cells infected with either Lm or Lm(ΔLLO) at each time point (#, below the level of detection).

An *in vitro* infection assay was employed to determine whether HRI regulates the intracellular proliferation of *L. monocytogenes*. Using this assay the initial invasion phase of the infection does not appear to differ between *Hri* +/+ and -/- cells (Figure **4A**; compare the ‘3’ hr time points). Following invasion, *L* *. monocytogenese* readily proliferates within *Hri* +/+ cells increasing ~5-fold after an additional 6 hrs of infection. In contrast, there is a relatively rapid and sustained decrease in the number of viable *L. monocytogenes* recovered from *Hri* -/- cells (Figure 4A). This latter infection profile (i.e., normal levels of invasion but defective post-invasion proliferation) is similar to that observed in *Hri* +/+ cells infected with an attenuated *L. monocytogenes* mutant strain lacking listerolysin O (LLO) (Figure 4A). This mutant strain invades cells but is quickly killed due to its inability to gain access to the cytosol [25]. Additionally, the infection profile of the attenuated LLO mutant strain was similar in *Hri* +/+ and -/- cells (Figure 4B) indicating that HRI is not required to rapidly eliminate this attenuated strain. These data show that the infection dynamics of virulent *L. monocytogenes* is greatly impacted by HRI.

**Figure 5:**
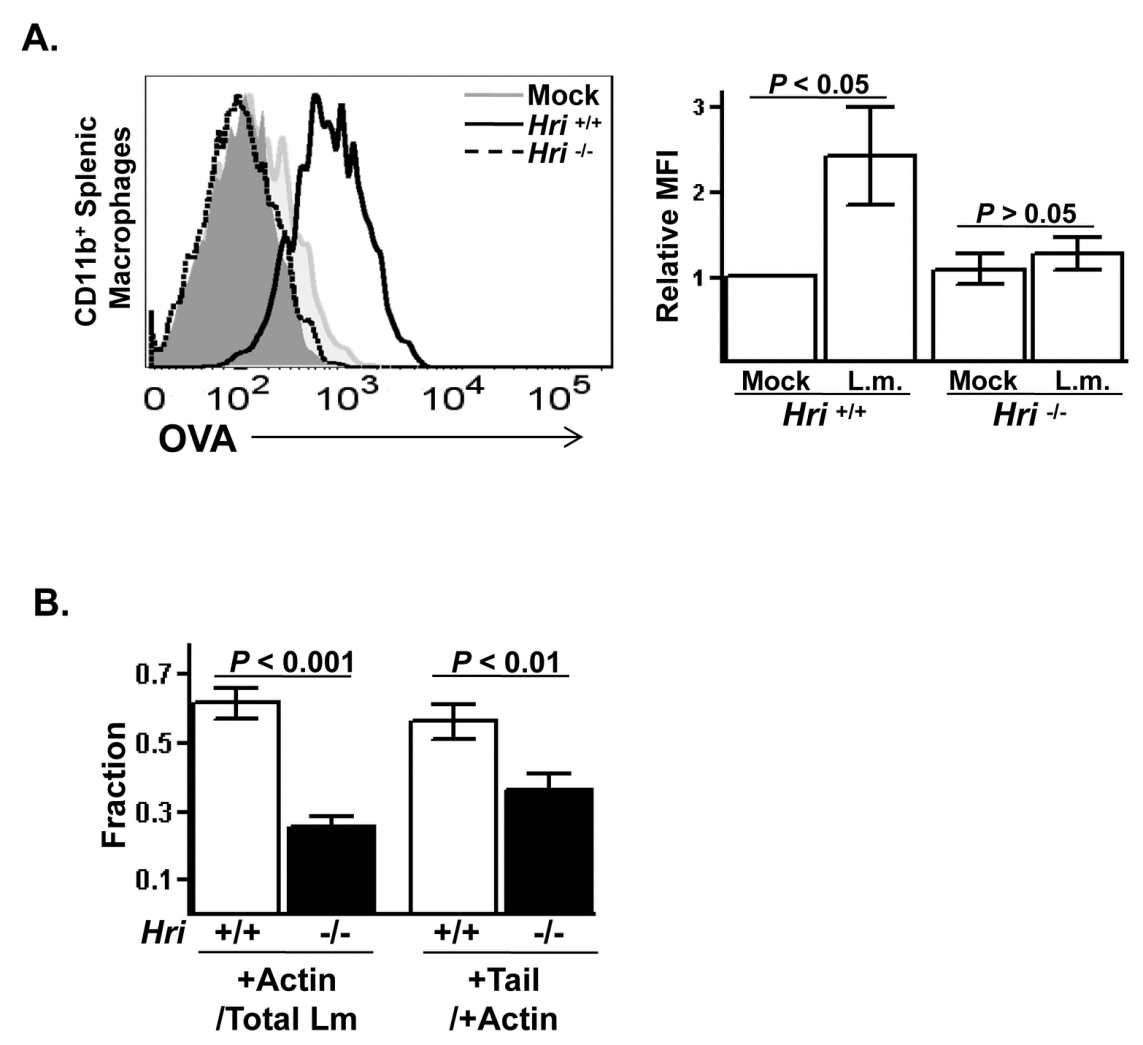
Absence of HRI reduces the translocation of *Listeria* into the cytosol. (**A**) Single-cell suspensions of splenocytes were prepared from *Hri* +/+ and -/- mice and either left uninfected or infected *in vitro* for 5 hrs with OVA-expressing *L. monocytogenes*. Histogram displays the levels of OVA surface staining on viable CD11b^+^-gated macrophages from a representative experiment and the relative median fluorescence intensities (MFI) of 3 separate experiments is shown in the right panel. The isotype control stained cells are shown in the grey filled plot. (**B**) Unstimulated peritoneal macrophages isolated from *Hri* +/+ and -/- mice were infected *in vitro* with GFP-expressing *L. monocytogenes* for 6 hr and then stained for actin. The fraction of *L. monocytogenes* (Lm) that were actin associated was determined by dividing the yellow and red-tailed green Lm by the total Lm (*Hri* +/+: N=418 Lm/168 macrophages; *Hri* -/-: N=542 Lm/167 macrophages). The fraction of cytosolic Lm that possessed actin tails was determined by dividing the red-tailed green Lm by the total number of yellow and red-tailed green Lm. (*P* values calculated using student *t* test.).

It was then tested whether HRI affects the delivery of *Listeria* -encoded factors to the cytosol. Splenocytes derived from *Hri* +/+ and -/- mice were infected *in vitro* with a *L. monocytogenes* strain expressing ovalbumin (OVA)-peptide. This peptide is a well-defined antigen that is loaded onto class I MHC molecules in the ER and presented on the surface of infected cells. Bacterial-derived OVA-peptide associated with MHC-I could readily be detected on *Hri* +/+ macrophages following a brief infection. In contrast, the MHC-I associated with OVA was not detected on *Hri* -/- macrophages (Figure 5A). These results suggest that *Listeria* -derived factors failed to access host cell cytosol in the absence of HRI.

Next we directly determined whether HRI is required for *L. monocytogenes* to gain access to the cytosol. Unstimulated peritoneal macrophages derived from *Hri* +/+ and -/- mice were infected *in vitro* with *L. monocytogenes*. There was no appreciable differences between *Hri* +/+ and -/- macrophages in the levels of internalized *L. monocytogenes* following brief infection period indicating that, like for the experiment shown in Figure **4** using fibroblastic cells, the invasion phase of *L. monocytogenes* infection is not regulated by HRI. However, differences between Hri +/+ and -/- cells started to become evident upon prolonged infection periods. Actin-associated *L. monocytogenes* (which serves as a marker for cytosolic bacteria) are first observed in *Hri* +/+ macrophages after 2 hr of infection in contrast to *Hri* -/- macrophages in which *L. monocytogenes* is exclusively found in vacuoles. After 6 hr of infection there was a significantly higher fraction of actin-associated *L. monocytogenes* in *Hri* +/+ macrophages compared to *Hri* -/- macrophages (Figure 5B). However, a substantial fraction of the bacteria that did make it to the cytosol in the *Hri* -/- macrophages were competent to form actin tails (Figure 5B) indicating that this post-escape phase of the infection cycle of *L* *. moncytogenes* is not regulated by HRI. These observations indicate that *L. monocytogenes* requires HRI to efficiently traffic to the cytosol.

To determine how HRI impacts *Listeria* -host cell interactions over longer infection periods, *Hri* +/+ and -/- cells were infected for 18 hrs and then stained for intracellular vacuoles and actin. There was a much higher level of general cytotoxicity in the infected *Hri* +/+ cells as evidenced by a greater number of detached and rounded cells. Among the surviving *Hri* +/+ cells many contained large numbers of *L. monocytogenes* (Figure **S4**). Additionally, the vast majority of *L. monocytogenes* in *Hri* +/+ cells were within the cytosol. By all measures, *Hri* -/- cells were much less impacted by long-term infection with *L. monocytogenes*. There was very little evidence of cytotoxicity in that infected cells maintained their original morphology and a substantial fraction of *L. monocytogenes* did not co-localized with actin (Figure **S4**, *right panels*). Collectively, three lines of evidence (bacterial proliferation, antigen processing, and direct observations) indicate that HRI specifically regulates the second phase of the *L. monocytogenes* cellular infection cycle: post-invasion trafficking to the cytosol.

## Discussion

Here we demonstrate that HRI positively affects the cell-level infection dynamics of three dissimilar bacterial pathogens. The extracellular pathogen *Yersinia* , the vacuole-bound pathogen *Chlamydia* , and the cytosolic pathogen *Listeria* , all require HRI to efficiently complete their respective cellular infection cycles. A common denominator among these three pathogens is that they all require access to the infected cell cytosol: either for their virulence factors to manipulate host cell processes ( *Yersinia* and *Chlamydia* ) or for the bacterium itself to reach the compartment in which it proliferates ( *Listeria* ).

How could a HRI-mediated process promote pathogen access to the host cytosol? A commonality among the three pathogens used in our studies is that their respective infection cycles are dependent on forming pores in infected host cellular membranes. In Gram-negative pathogens such as *Yersinia* and *Chlamydia* , the T3S secrete translocators that assemble pore-forming structures in the host plasma membrane that mediate the transfer of effectors into the cytosol [14,17]. In some respects this process resembles that which occurs in *Listeria* -infected cells. Following its invasion of the host cell, *Listeria* secretes monomeric LLO that, following its activation by the host-encoded gamma-interferon-inducible lysosomal thiol reductase (GILT) [26], binds to and oligomerizes into pore-forming structures within the endosomal membrane. In addition to allowing leakage of antimicrobial factors, the resulting pores are also thought to allow the access of co-expressed and secreted phospholipases to the inner leaflet of the endosomal membrane [24,25]. It is possible that one or more of these events occur with reduced efficiency in HRI null cells.

We believe that the activities of HRI described here neither involve it acting as an eIF2α kinase nor otherwise affecting protein synthesis. That T3S secretion is not coupled to host cell protein translation was indicated by our finding that cycloheximide treatment did not affect YopE-mediated disruption of the host cell cytoskeleton. The most compelling data, in our opinion, supporting the model that the infection-specific activities of HRI are independent of its role as a regulator of protein synthesis is the fact that cells lacking the Ser51 residue of eIF2α (the phosphorylation site of HRI and the other eIF2α kinases) are just as competent as wild-type cells in supporting both the T3S activity of *Yersinia* as well as the T3S-dependent intracellular proliferation of *Chlamydia* [4]. However, eIF2α(S51A)-expressing cells are more permissive for bacterial invasion indicating that eIF2 signaling does impact the initial events of the pathogen–host cell interaction. For example, *Chlamydia* is much more efficient at forming inclusions in eIF2α(S51A) cells; however, the number of infectious EBs per inclusion (a measure of intra-vacuolar growth) is comparable between wild-type and eIF2α(S51A) cells [4]. This latter phenotype resembles that observed for PKR null cells (Figure 3) indicating that PKR-mediated eIF2 signaling acts to oppose bacterial invasion but does not affect the subsequent maturation of the Chlamydial inclusion. Our findings are consistent with the observation first noted by Alexander [27] of increased Chlamydial proliferation in cycloheximide-treated cells due to enhanced pathogen invasion.

Owing to its broad activity in promoting the intracellular proliferation of pathogens, HRI may be an excellent target for the development of anti-microbial compounds. HRI is an especially attractive target since it is not required for responses to non-pathogens but interferes with specific virulence mechanisms. Recently, it has been shown that HRI activity can be reduced by either direct targeting with small molecules or indirectly by using natural products that inhibit the HRI-cofactor Hsp90 [28–30]. The targeting of host factors may make it less likely that a pathogen would evolve drug-resistance since the pathogen would not have genetic control over the interaction between the compound and its target.

## Supporting Information

Figure S1 Comparable adhesion of *Y. pseudotuberculosis* to *Hri* **+**/**+** and -/- cells. The indicated MEFs were infected with *Y. pseudotuberculosis* at a MOI of 5 for 45 min. Cells were then washed several times, lysed, and the resulting lysates were plated on LB media. Two days later the resulting colonies were enumerated and plotted is the average number of colonies from three independently-infected wells.
(TIFF)

Figure S2 Cycloheximide does not affect the type 3 secretion system of *Y. pseudotuberculosis*. HeLa cells were treated were either treated or not with cycloheximide (25 µg/ml) one hour prior to the addition of *Y. pseudotuberculosis* as well as for 2 additional hours of infection at which time live cells were imagined.
(TIF)

Figure S3 Delayed fusogenecity of *C. trachomatis* in *Hri* -/- cells. MEFs were infected at an MOI of 5-10 and visualized 20 hrs post infection. On average one inclusion per cell was detected in *Hri* +/+ while multiple inclusions per cell routinely occurred in *Hri* -/- cells.
(TIF)

Figure S4 *Hri* -/- cells are highly resistant to long-term *Listeria* infection. *Hri* +/+ and -/- MEFs were infected with GFP-expressing Lm for 18 hrs and then stained for actin (red), nuclei (purple), and vacuoles (pink). Shown in the enlarged images are numerous actin-associated Lm in *Hri* +/+ cells and in the *Hri* -/- cells either non-actin associated Lm (green) or Lm that are lightly associated with actin (yellow).
(TIFF)
