# Supplementary figures and images for: The Host-Encoded Heme Regulated Inhibitor (HRI) Facilitates Virulence-Associated Activities of Bacterial Pathogens
Source: PLoS One. 2013 Jul 10;8(7):e68754. doi: 10.1371/journal.pone.0068754 (PMC3707855; doi:10.1371/journal.pone.0068754)

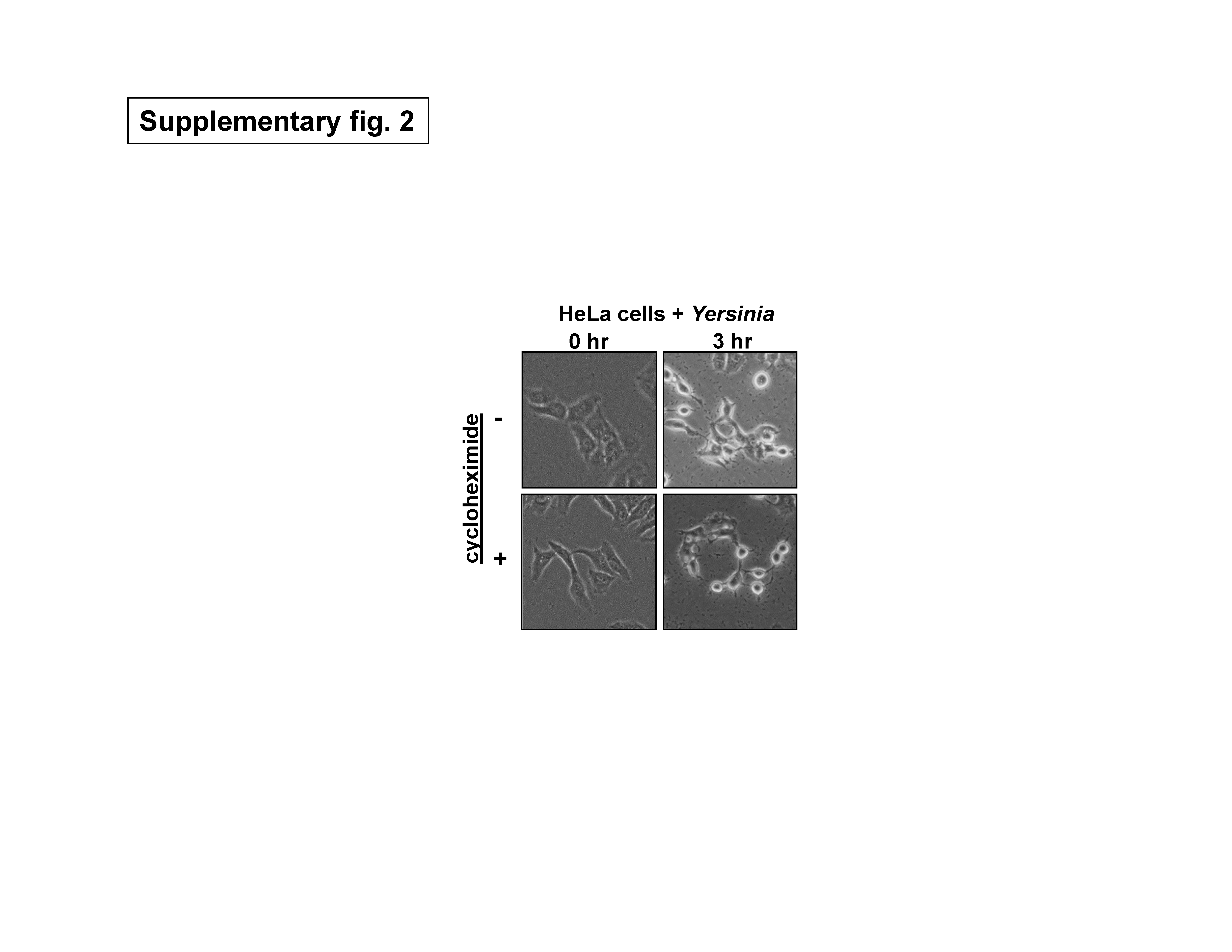

Supplement: Figure S2 — HeLa cells were treated were either treated or not with cycloheximide (25 µg/ml) one hour prior to the addition of Y. pseudotuberculosis as well as for 2 additional hours of infection at which time live cells were imagined. (TIF) [file pone.0068754.s002.tif]

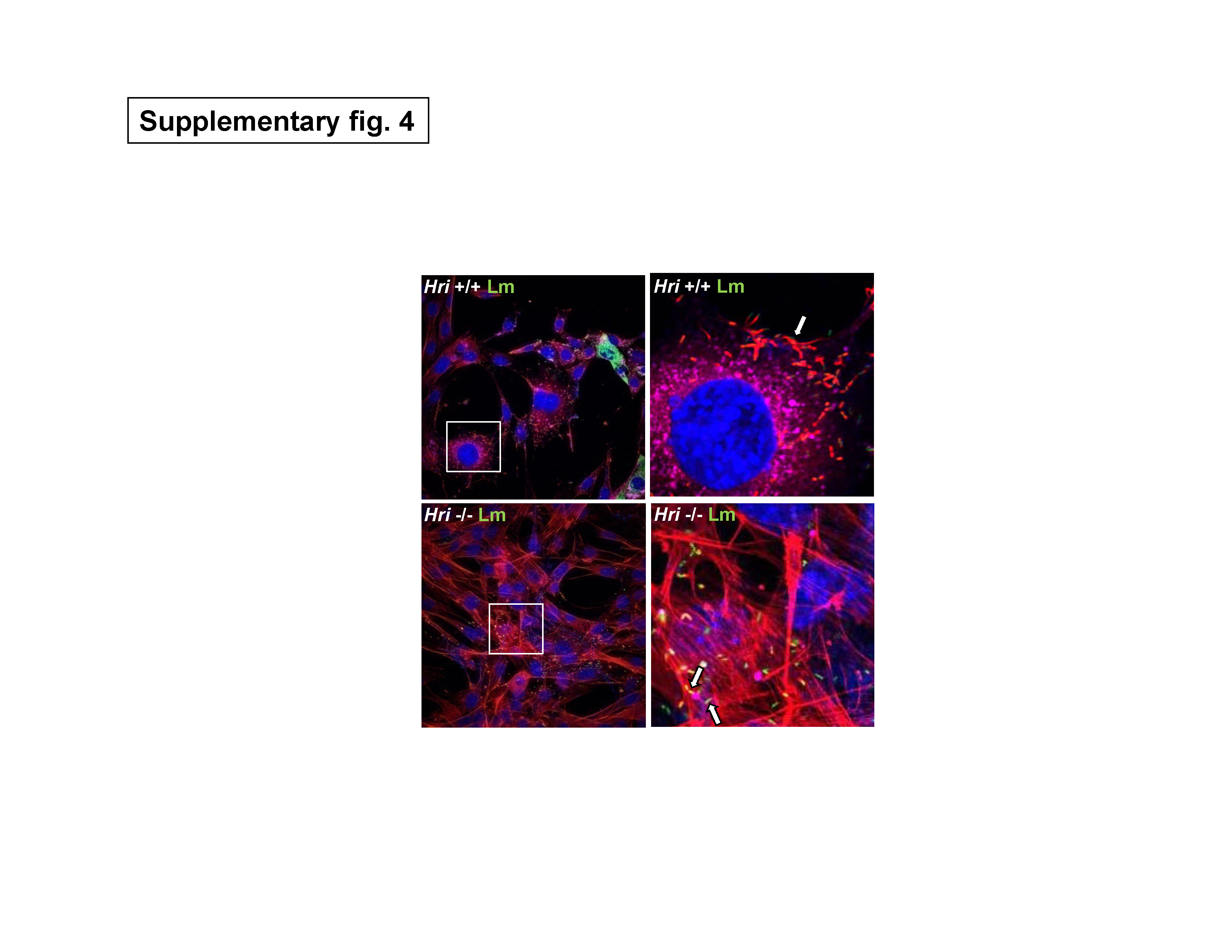

Supplement: Figure S4 — Hri +/+ and -/- MEFs were infected with GFP-expressing Lm for 18 hrs and then stained for actin (red), nuclei (purple), and vacuoles (pink). Shown in the enlarged images are numerous actin-associated Lm in Hri +/+ cells and in the Hri -/- cells either non-actin associated Lm (green) or Lm that are lightly associated with actin (yellow). (TIFF) [file pone.0068754.s004.tiff]
